# The impact of agency on time and risk preferences

**DOI:** 10.1038/s41467-020-16440-0

**Published:** 2020-05-29

**Authors:** Ayelet Gneezy, Alex Imas, Ania Jaroszewicz

**Affiliations:** 1 UC San Diego, Rady School of Management, 9500 Gilman Dr., La Jolla, CA 92093 USA; 2 Carnegie Mellon University, Social & Decision Sciences, 5000 Forbes Ave., Pittsburgh, PA 15213 USA; 3 Harvard University, The Institute for Quantitative Social Science, 1737 Cambridge St., Cambridge, MA 02138 USA

**Keywords:** Decision making, Economics

## Abstract

Scholars have long argued for the central role of agency—the size of one’s choice set—in the human experience. We demonstrate the importance of agency in shaping people’s preferences. We first examine the effects of resource scarcity—which has been associated with both impatience and a lack of agency—on patience and risk tolerance, successfully replicating the decrease in patience among those exposed to scarcity. Critically, however, we show that endowing individuals with agency over scarcity fully moderates this effect, increasing patience substantially. We further demonstrate that agency’s impact on patience is partly driven by greater risk tolerance. These results hold even though nearly all individuals with greater agency do not exercise it, suggesting that merely knowing that one could alleviate scarcity is sufficient to change behavior. We then demonstrate that the effects of agency generalize to other adverse states, highlighting the potential for agency-based policy and institutional design.

## Abstract (web summary)

Scholars have long argued for the central role of agency—the size of one’s opportunity set—in the human experience, but there has been little work on how a sense of agency affects behavior. We demonstrate that increasing agency leads to greater patience and risk tolerance, even if these new opportunities are not exercised.

## Introduction

The importance of personal agency has been discussed as early as Aristotle’s *Nicomachean Ethics*. Prominent philosophers (ref. ^1^), political scientists^2^, and economists across the ideological spectrum—from Marx^3^ to Friedman^4^—have argued for the central role of agency in the human experience. Defined in relation to an individual’s choice set, where a larger set of opportunities is associated with greater agency^5^, the value of personal agency has been attributed to two main sources. Economists have primarily focused on the purely instrumental value of agency: a larger choice set increases the potential to select an option that leads to greater utility^4^. Philosophers and psychologists have argued for the intrinsic value of personal agency, positing that people may value greater agency as an end in itself^6–9^. Indeed, individuals whose agency has been diminished seek to punish those deemed responsible^10^ and score higher on measures of well-being when it is restored^11,12^—even when the increased agency carries no pecuniary benefits.

This paper focuses on the important role of personal agency along a third dimension: shaping people’s preferences. Specifically, we examine how agency over one’s environment affects individuals’ level of patience and tolerance for risk. Our results show that increasing personal agency increases risk tolerance and leads to a greater willingness to wait for larger, later rewards over smaller, sooner rewards. Importantly, we show these effects hold even though the vast majority of individuals in our sample do not exercise their greater agency. This suggests that simply knowing one has greater agency is sufficient to impact behavior, even if that additional agency is not used. Our results carry significant implications for the role of agency in decisions involving tradeoffs over time and risk, such as whether to save for the future, get more schooling, or adopt and maintain a healthier lifestyle.

Why would agency affect patience and tolerance for risk? Prior work has shown that compared to people with a relatively low sense of agency, those with a greater agency perceive prospects as less risky, believing that negative outcomes are less likely to occur^13,14^. These findings imply that people with a perception of greater agency will be more willing to take on risk, compared to those with less agency^15^. Such changes in risk preferences have implications for intertemporal choice^16^. Decisions over time inherently involve a tradeoff between different levels of risk, since the more distant future is less certain than dates closer to the present^17^. Researchers^18^ have argued that this difference in uncertainty can at least partially explain myopia and impatience: if delayed rewards are more uncertain than sooner rewards, then people who are more averse to risk should prefer smaller, sooner rewards to larger, later ones. Consequently, if agency increases risk tolerance, individuals with greater agency will be more patient and prefer larger, later rewards.

To motivate our investigation of the relationship between personal agency and decision-making, we use the 2010–2014 World Values Survey multinational dataset^19^ (*N* = 86,272; 61 countries). We test whether higher levels of reported agency are associated with higher reported savings, which researchers have argued is a manifestation of greater patience^20,21^. As part of the survey, participants are asked whether, in the last year, their family had saved money, just gotten by, spent some savings, or spent savings and borrowed money. We use these responses to capture reported savings. To capture perceived agency, we use responses to the question, “Some people feel they have completely free choice and control over their lives, while other people feel that what they do has no real effect on what happens to them. Please use this scale where 1 means ‘none at all’ and 10 means ‘a great deal’ to indicate how much freedom of choice and control you feel you have over the way your life turns out.” See Supplementary Method 1.

Our analyses show that even when controlling for a host of explanatory variables such as income, employment, marital status, and education, people’s reported levels of agency explain a significant proportion of their savings behavior: people who report a greater sense of agency save significantly more (ordinary least squares [OLS] regression with robust standard errors [SEs], clustering at the country level, *p* = 0.001). This relationship between respondents’ sense of agency and savings behavior is robust to different model specifications (see Supplementary Table 1) and holds for both Organization for Economic Cooperation and Development (OECD) and non-OECD countries. Importantly, we further test whether agency can explain residual variation in reported savings that cannot be captured by standard economic determinants considered in the literature. We first regress savings on employment, income, and a multitude of other explanatory variables, using robust SEs and clustering at the country level. We then regress the residuals from this model on agency. Supplementary Fig. 1 illustrates the significant positive relationship between the unexplained variation in savings and individuals’ reported agency (*p* < 0.0005), showing the relationship both by country (left panel) and collapsing across countries (right panel). Because these results come with the usual caveats associated with using correlational data, we do not claim they imply causality. Rather, we use them as initial motivating evidence for our investigation.

This paper reports results from two experiments that demonstrate the proposed causal effects of agency on behavior. In both studies, participants were placed in an aversive environment and endowed with different levels of agency over it: some had the opportunity to alleviate their state, whereas others did not. In the first study, participants faced resource scarcity. Specifically, they were asked to work on a task but lacked enough time to complete it. Participants who had the option to alleviate the scarcity—to increase the time allotted to the task—were substantially more patient on a subsequent task than those who lacked it: they were significantly more likely to prefer a larger, later financial reward to a smaller, sooner one. These results hold despite the fact that the vast majority of participants did not exercise their agency (i.e., they did not choose to alleviate the scarcity), suggesting that merely knowing that one has the option to improve one’s circumstances is sufficient to promote greater patience. In fact, the choices made by participants who had agency were indistinguishable from those made by participants who did not face scarcity at all. In addition, in line with our theoretical framework, our analyses reveal that the relationship between agency and patience is mediated by shifts in risk tolerance—a lack of agency decreases willingness to take on risk, which, in turn, decreases patience.

Our second study tested whether the effect of agency on behavior can be generalized to other settings—specifically, exposure to environmental stressors. Participants were asked to complete a task while being exposed to aversive noise. Some had agency over this state—an option to alleviate the noise—whereas others did not. As in the first study, we find that people who had greater agency were subsequently more likely to wait for a larger, later reward than to select a smaller, sooner one. Again, this increase in patience occurred despite the fact that the vast majority of participants did not exercise their agency, meaning they were exposed to the same level of environmental stressor as our No Agency participants.

Our findings contribute to the literature on the effects of adverse states, such as scarcity, on preferences and behavior. Resource scarcity has been linked with greater risk aversion^22^, greater discounting of the future^23,24^, and a greater propensity to engage in behaviors involving immediate rewards and delayed costs, such as smoking^25^. Researchers have proposed that this shift in behavior is driven by the experience of scarcity itself^26,27^. Experiencing scarcity has been shown to drive greater risk aversion^28^ and lead to less patient decisions^29,30^. However, prior work suggests those experiencing adverse states, such as poverty, also report a pronounced lack of agency over their state^31^. And indeed, participants in prior studies who were placed in a state of scarcity often lacked agency over that condition: they had no option to alleviate the state. This coexistence of adversity and lack of agency makes it difficult to identify the extent to which the state of scarcity per se*—*rather than the lack of agency over the state—drove the observed effects. Our findings suggest that agency may, in fact, play a key role in the previously documented relationship between adverse states and impatience: increasing perceived agency among those facing adverse states seems to mitigate the behavioral effects of experiencing the state.

These results are pertinent in light of recent findings documenting the success of poverty-alleviation programs offering unconditional cash transfers to people living in poverty (e.g., GiveDirectly). These programs have been shown, for instance, to significantly improve outcomes such as schooling and physical health^32^. Rather than limiting individuals’ options like the more traditional in-kind benefit transfers, unconditional cash transfers allow recipients to choose exactly how to spend their money, thereby increasing their personal agency. Our findings suggest that this increase in agency may not only have instrumental and/or intrinsic benefits—it may also lead recipients to use the funds in a more forward-looking manner.

## Results

### Study 1

Our first study tested whether being endowed with agency over resource scarcity increases patience and tolerance for risk. Participants were randomly assigned to one of three conditions: Scarcity–Agency (hereafter Agency), Scarcity–No Agency (hereafter No Agency), and a condition without scarcity (hereafter Control). While participants in the Control group had enough time to complete a task (answering a series of cognitive aptitude questions), those in the scarcity conditions faced significant time contstraints. Participants in both scarcity conditions (No Agency and Agency) faced the same levels of scarcity; however, those in the Agency condition had the option to alleviate their scarcity anytime—they could press a button to gain more time to complete the task. To discourage this agency option from actually being exercised, we imposed a substantial cost to doing so. This allowed us to compare two groups that faced the same level of scarcity, but differing levels of agency.

Next, participants made two choices. For each choice, they allocated tokens across an earlier and a later date, where tokens allocated to later dates would be worth more money than tokens allocated to earlier dates. The more tokens allocated to the later dates, the more money the participant stood to earn. This served as our measure of patience. We measured risk preferences by asking participants to make four binary choices between safer and riskier financial gambles. We also included several manipulation checks. The Methods section includes a detailed description of the procedures and measures used.

Drawing on prior findings from the scarcity literature, we predicted that those who faced scarcity but lacked agency over it would be more impatient than those in the Control condition. We further hypothesized that having greater agency would mitigate this increased impatience, such that despite facing the same level of scarcity, participants in the Agency condition would allocate more tokens to the earlier dates than those in the No Agency condition. Finally, if a lack of agency affects patience through a shift in risk tolerance, the observed differences in patience should be mediated by differences in risk tolerance.

Only four of the 69 participants in the Agency condition chose to exercise their agency and gain more time. The remaining 65 participants faced the same time constraints as participants in the No Agency condition. In addition, two participants in the Control condition did not complete the experiment, exiting before finishing the second set of cognitive aptitude questions.

Manipulation checks confirmed that both our scarcity and agency manipulations were effective at producing the intended states. With respect to the former, participants in both scarcity conditions answered, on average, approximately four fewer cognitive aptitude questions correctly than those in the Control condition (*M*_ScarcityGroups_ = 8.17; *M*_Control_ = 12.28; two-tailed pairwise *t* test, *t*(211) = 11.715, *p* < 0.0005). Participants in the two scarcity conditions answered approximately the same number of questions (*M*_Agency_ = 8.31; *M*_NoAgency_ = 8.05; two-tailed pairwise *t* test, *t*(139) = 0.686, *p* = 0.49). This allows us to rule out ego depletion^33^ and differences in cognitive function as alternative explanations for our results. Participants in both scarcity conditions also reported feeling significantly more time-constrained (*M* = 2.38) than those in the Control condition (*M* = 4.59; two-tailed pairwise *t* test, *t*(209) = 13.056, *p* < 0.0005), providing additional evidence that our manipulation was effective in generating scarcity (see Supplementary Table 2). Supplementary Fig. 3 and Supplementary Table 5 further show our scarcity manipulation affected both the amount of time participants took to complete the task, as well as the number of questions left unanswered.

Participants in the No Agency condition reported significantly lower perceived agency (*M* = 2.30) than those in the Control condition (*M* = 2.74; two-tailed pairwise *t* test, *t*(144) = 2.087, *p* = 0.039), while reported agency of participants in the Agency (*M* = 2.51) and Control conditions did not differ (two-tailed pairwise *t* test, *t*(133) = 1.103, *p* = 0.272), suggesting our agency manipulation was effective. Consistent with our proposed framework, there were no significant differences between the Agency and No Agency conditions in the three affect measures—anger, sadness, and happiness (two-tailed pairwise *t* tests, all *p*s ≥ 0.136).

**Fig. 1:**
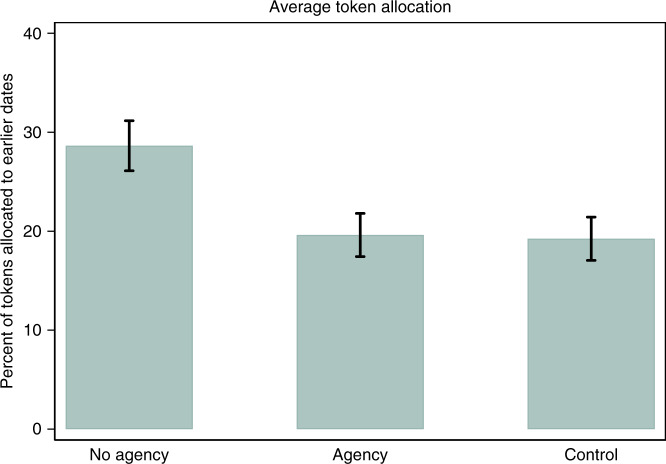
Study 1 results: percent of tokens allocated to earlier dates, collapsing across allocation decisions. *N* = 211 participants. Error bars denote mean values ± 1 SE.

**Fig. 2:**
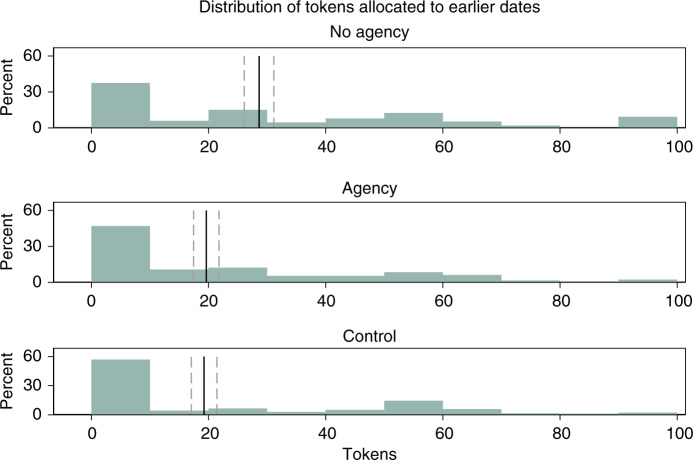
Study 1 results: percent of tokens allocated to earlier dates, collapsing across allocation decisions. *N* = 211 participants. Black solid line denotes mean; gray dashed lines denote mean value ± 1 SE.

The proportion of tokens allocated to the earlier date did not differ between the two allocation decisions for any of the three conditions: participants made approximately the same allocation decisions when choosing between “today” and “one week from today” as when choosing between “one week from today” and “two weeks from today” (two-tailed pairwise *t* tests, *p* ≥ 0.077 for all three groups when comparing within group; *p* = 0.136 when collapsing across groups). As is standard in the time preference literature^34,35^, we collapse across the two allocation decisions and use the number of tokens allocated to each of the two earlier dates as our primary dependent variable. Figures 1 and 2 show token allocations to the two earlier dates by condition. Supplementary Fig. 2 presents results for each allocation decision separately.

First, we find that participants with greater agency were substantially more patient than those lacking agency, despite the fact they faced the same level of scarcity. Participants in the Agency condition allocated about 30% fewer tokens to earlier dates than those in the No Agency condition, thus capitalizing on the larger rewards associated with the later dates (*M*_Agency_ = 19.6; *M*_NoAgency_ = 28.6; OLS regression with robust SE clustered at the participant ID level with Agency as the omitted group, *p* = 0.041). Second, our analyses show that endowing participants with agency over scarcity made their choices indistinguishable from those who did not face scarcity at all: participants in the Agency and Control conditions allocated essentially the same number of tokens to the earlier dates (*M*_Control_ = 19.2; OLS regression with robust SE clustered at the participant ID level with Control as the omitted group, *p* = 0.924). Third, consistent with the previous literature, we find that those in the No Agency condition were also substantially less patient than those in the Control condition (OLS regression with robust SE clustered at the participant ID level with Control as the omitted group, *p* = 0.031) (see Table 1). These results support our proposition that perceived agency moderates the effect of scarcity on patience.

**Table 1:** Study 1 results.

	(1)	(2)
No Agency	9.0**	9.4**
	(4.4)	(4.5)
Control	−0.4	−1.8
	(4.0)	(6.2)
Second token decision	2.1	2.1
	(1.5)	(1.5)
Constant	18.6***	19.3**
	(3.0)	(8.9)
Additional covariates?	No	Yes
*N*	421	421
*R*^2^	0.027	0.038
*p* Value	0.035	0.068

Outcome variable: number of tokens allocated to earlier dates. Ordinary Least Squares regressions with robust standard errors clustered at the participant ID level. Agency is the omitted category. Each participant appears in the regression twice: once for each of the two time-preferences token allocation decisions. *Second token decision* is an indicator variable controlling for the token allocation decision. Additional covariates are the participant’s score for the first set of cognitive aptitude questions, their score for the second set of cognitive aptitude questions, and their responses to the question on perceived time scarcity. Column (1) No Agency: *p* = 0.041. Column (2) No Agency: *p* = 0.038. Standard errors are in parentheses.

*p < 0.10, **p < 0.05, ***p < 0.01

**Fig. 3:**
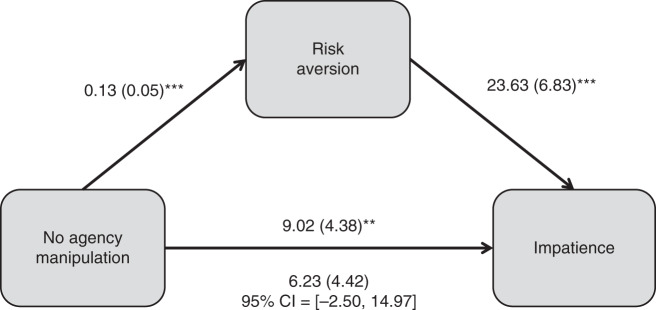
Study 1: the effect of lack of agency on impatience, as mediated by risk aversion. All statistics compare only the two scarcity groups and use OLS with robust SE clustered at the participant ID level. Regression coefficients are unstandardized. Standard errors are in parentheses. For the pathway from *No Agency manipulation* to *Risk Aversion*: *p* = 0.006; for the pathway from *Risk Aversion* to *Impatience: p* = 0.001. For the pathway from *No Agency manipulation* to *Impatience*, the values above the arrow stem from the model without the mediator (*p* = 0.041), whereas the values below the arrow stem from the model with the mediator (*p* = 0.160). *p < 0.10, **p < 0.05, ***p < 0.01.

Having demonstrated the relationship between agency and patience, we next examined the role of risk tolerance in explaining this relationship. We measure risk tolerance by creating a variable corresponding to the proportion of times the participant chose the safer option over the riskier one in the four binary risk questions, with greater values corresponding to greater risk aversion. Comparing the two scarcity conditions, we use OLS with robust standard errors to regress the risk-tolerance variable on a dummy variable for the No Agency condition. This analysis reveals that individuals with greater agency over scarcity were significantly less likely to choose the safe option than those lacking agency (*B* = 0.13; *p* = 0.006). Regressing token allocation on both the experimental-condition dummy and the risk variable shows that participants’ responses to the risk measures largely explain the observed relationship between agency and token allocation. In particular, while lower risk tolerance is associated with allocating a greater number of tokens to earlier dates (OLS with robust SE clustered at the participant ID level when including only scarcity groups: *B* = 23.63; *p* = 0.001; OLS with robust SE clustered at the participant ID level when including all three conditions: *B* = 20.37*; p* < 0.0005), the coefficient on the No Agency experimental condition dummy changes from being significant when the risk-tolerance variable is not included (OLS with robust SE clustered at the participant ID level, *B* = 9.02; *p* = 0.041) to being nonsignificant when it is included (OLS with robust SE clustered at the participant ID level, *B* = 6.23; *p* = 0.160). Approximately 30% of the effect generated by a lack of agency on patience can be attributed to the indirect effect on risk tolerance. Figure 3 summarizes these findings.

To ensure that all participants in our two main conditions of interest—Agency and No Agency—experienced the same level of adversity, we had discouraged participants from exercising their agency and excluded participants who nonetheless decided to do so. This particular feature of our experimental design introduces a potential self-selection issue: the Agency participants in our samples include only those who chose not to exercise their agency. To ensure that our results are robust to including the small fraction of participants (4 out of 69 (6%)) who chose to exercise their agency, we calculated Lee bounds^36^, which offer a conservative estimate of our treatment effect. The results of this analysis reveal that the 95% confidence interval for the treatment effect does not include 0 for either the patience (0.32–16.91) or risk tolerance (0.04–0.25) measures when testing the difference between the Agency and No Agency conditions using our primary regression specification. Our results are also robust to controlling for the time preferences elicitation comprehension check responses and to using a different operationalization of risk preferences. These analyses are reported in Supplementary Note 1. In addition, in Supplementary Note 2, we describe a replication study where all participants faced the same time constraint for the first set of cognitive aptitude questions, and those in the control condition faced less (as opposed to no) scarcity in the second set of cognitive aptitude questions.

Lastly, to further demonstrate that our manipulation affected agency and ensure that the observed differences in Study 1 were not driven by changes in other constructs (i.e., negative affect and trust in the experimenter), we ran an additional study (Study 1b). There, we randomly assigned participants to one of the three same conditions used in Study 1, then used a behavioral measure to capture subsequent demand for agency. We also elicited participants’ emotional state and their trust in the experimenter. The results support our hypothesis that the manipulation affected participants’ sense of agency but did not influence their negative affect or trust in the experimenter. Those who experienced scarcity but could gain more time had a lower subsequent demand for agency than those who lacked this option. In addition, there were no significant differences in negative affect or trust in the experimenter between any of the groups. Supplementary Method 3 and Supplementary Note 3 describe the experimental design and results for this study, respectively. One potential limitation of Study 1 is that the negative impact of the adverse state, and thus our agency manipulation, could have been greater for those with low cognitive ability. Although random assignment ensures our conclusions about the effects of agency on behavior are still valid on average, we cannot rule out this possibility.

### Study 2

The results of Study 1 provide direct evidence for our hypotheses: greater agency increases tolerance for risk, which in turn leads to greater patience. In Study 2, we examined whether the observed relationship between agency and patience generalizes to other contexts. Specifically, we tested whether greater agency can moderate the effect of environmental stressors on time preferences.

All participants were instructed to complete a task (solve a series of anagrams) while listening to a loud noise through headphones. Participants in the No Agency group were not allowed to remove these headphones, while those in the Agency group were given this option. As in Study 1, participants in the Agency group could only exercise their agency if they incurred a significant cost. This ensured that the two groups faced the same level of environmental stressors and varied only insofar as the Agency group had the option of alleviating the stressor. After completing the task, participants made a series of choices between a smaller, sooner reward and a larger, later reward, which served as a measure of their patience. The Methods section provides a detailed description of the procedures and measures used.

We predicted that participants with greater agency—those who had the option to remove their headphones—would exhibit greater patience relative to those who did not have this option. Specifically, we hypothesized that participants in the Agency condition would be more willing to wait for larger, later rewards than those in the No Agency condition, despite being exposed to the same level of environmental stressors.

Two participants (both in the Agency condition) removed their headphones during the experiment. To ensure all participants included in our analysis experienced the same adverse stimuli, we exclude these participants from the main analysis. This leaves 113 participants (*N*_NoAgency_ = 54; *N*_Agency_ = 59). Participants in both conditions solved, on average, a similar number of anagrams (*M*_NoAgency_ = 6.24 versus *M*_Agency_ = 5.17; two-tailed pairwise *t* test, *t*(111) = 1.525, *p* = 0.130).

**Fig. 4:**
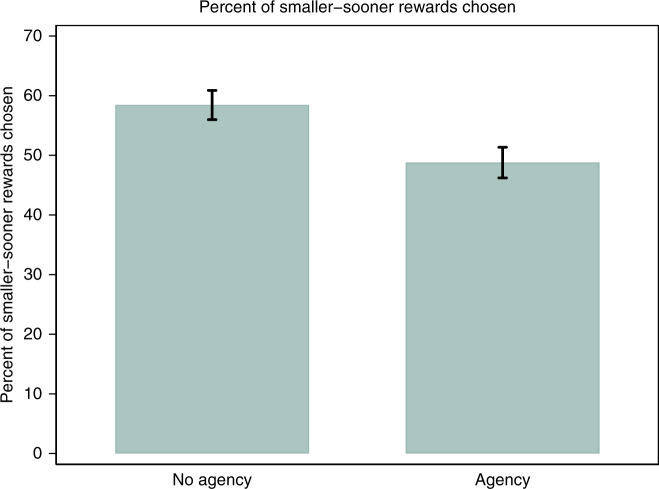
Study 2 results: percent of smaller-sooner rewards chosen, by treatment group. *N* = 113 participants. Error bars denote mean values ± 1 SE.

**Fig. 5:**
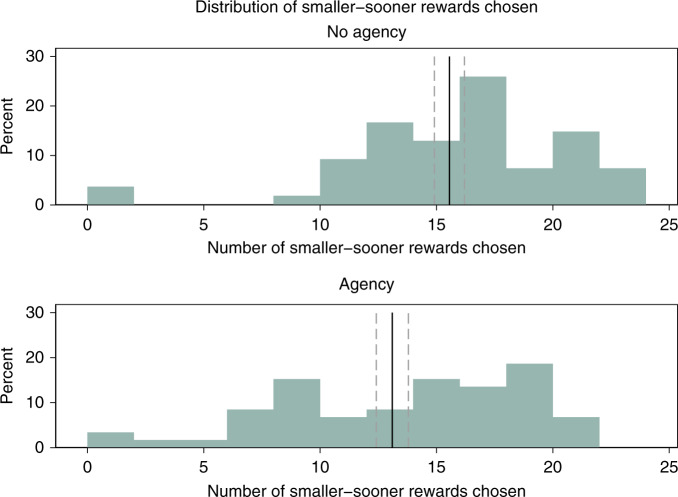
Study 2 results: distribution of smaller-sooner rewards chosen, by treatment group. *N* = 113 participants. Black solid line denotes mean; gray dashed lines denote mean values ± 1 SE.

Despite being exposed to the same level of noise, participants in the Agency condition made significantly more patient choices than those in the No Agency condition. In particular, they chose the smaller, sooner reward on average 49% of the time, whereas those in the No Agency condition chose it 58% of the time (OLS with robust SE, *p* = 0.008; Mann–Whitney *U* test, *p* = 0.015) (see Figs. 4 and 5).

To verify that our results are robust to including the two participants who chose to exercise their agency, we conduct an intent-to-treat analysis. The analysis reveals that our conclusions are unchanged when including these participants (all *ps* ≤ 0.018) (see Table 2).

**Table 2:** Study 2 results.

	(1)	(2)	(3)
Agency	−9.7***	−9.6***	−10.2***
	(3.6)	(3.6)	(3.1)
Anagrams solved	−0.1	0.0	0.0
	(0.5)	(0.4)	(0.4)
Removed headphones	−0.8		
	(34.7)		
Constant	58.8***	58.4***	60.6***
	(3.7)	(3.7)	(3.3)
Includes Ps who removed headphones	Yes	No	No
Includes Ps who have non-rationalizable time preferences	Yes	Yes	No
*N*	115	113	109
*R*^2^	0.057	0.062	0.092
*p* Value	0.069	0.029	0.005

Outcome variable: percent of smaller-sooner rewards chosen. Ordinary Least Squares regressions with robust standard errors. Column (1) Agency: *p* = 0.009. Column (2) Agency: *p* = 0.009. Column (3) Agency: *p* = 0.001. Standard errors in parentheses.

*p < 0.10, **p < 0.05, ***p < 0.01.

Next, we used participants’ responses to the 27 time-preference questions to identify each individual’s indifference point between smaller, sooner rewards and larger, later ones. This analysis allowed us to estimate the discount parameter *k* of the hyperbolic discounting formula, which has previously been used to fit intertemporal choice data^37^.

1 $$V = \frac{A}{{1 + kD}}$$

Here, *D* is the delay in days, *A* is the delayed reward, and *V* is the present value of the delayed reward. The indifference point provides the values of the present gain *V* that makes an individual indifferent between receiving *V* now or a delayed gain *A* at point *D* days in the future. Using these data points, we can solve for the discounting parameter *k*, where a larger *k* corresponds to greater myopia and impatience^35,38,39^.

For this analysis, we had to exclude four participants (two from each condition) who chose either the first (smaller-sooner) or second (larger-later) reward across all 27 time-preferences questions, as such responses yield non-rationalizable time preferences. This leaves us with 109 participants (*N*_*NoAgency*_ = 52; *N*_*Agency*_ = 57). The mean discounting parameter *k* of participants in the Agency condition (*M* = 0.007) was significantly smaller than that of participants in the No Agency condition (*M* = 0.015; two-tailed pairwise *t* test, *t*(107) = 2.727, *p* = 0.008). These results are consistent with the nonparametric results, providing further support for our proposition that endowing participants with agency over an adverse state leads to significantly more patient choices.

We ran an additional study (Study 2b) as a manipulation check testing the extent to which the agency manipulation used in Study 2 successfully influences participants’ perceived level of agency. For our main dependent variable, we used a behavioral measure of demand for agency similar to the one we used in Study 1b. The results support our hypothesis that our agency manipulation was successful at shifting participants’ sense of agency: participants who had the option to remove the headphones had a lower subsequent demand for agency than those who did not have this option. The design and results for this study are described in Supplementary Method 5 and Supplementary Note 4, respectively.

## Discussion

In this paper, we show that personal agency over one’s environment has a significant impact on preferences. We first show that people who have agency over resource scarcity make more patient decisions than those who lack agency over the same scarcity (Study 1). In fact, people with agency were as patient as those who did not experience scarcity at all. We observe this effect despite the fact that the vast majority of participants did not exercise their agency at all. Our results suggest that simply knowing one has greater agency is sufficient to affect individuals’ preferences. Further, we show that the effects of agency on intertemporal choice are driven by changes to individuals’ risk tolerance. Considered in the context of recent work examining the effects of scarcity on decision-making, our findings point to a distinct pathway through which exposure to resource scarcity may affect behavior—namely, through the channel of personal agency^27,30^.

In Study 2, we demonstrate that the effects of agency on patience extend to other adverse states such as exposure to environmental stressors. Here, people who had agency over an aversive noise were more likely to make patient decisions than those who lacked the option, despite the fact that the vast majority did not utilize this option and experienced the same level of noise.

It is worth noting that in Study 1, we did not find evidence for present bias (greater impatience across sooner dates than later dates) among any of the three groups. These results are consistent with recent work that does not find evidence for present bias when the first payment is delayed by even a short amount of time, as it is in our setting^34^. Our results are also consistent with recent work documenting that higher community trust can decrease temporal discounting among low-income individuals^40^. To the extent that trusting one’s community to ease one’s liquidity constraints provides one with a greater sense of agency over one’s poverty, our results suggest a possible pathway through which such trust may result in greater patience.

Our findings have implications for policies aimed at alleviating adverse states such as poverty, prescribing an approach that differs substantially from those implied by existing theories. For example, the structural theory of poverty^41^ argues that one could mitigate persistent poverty by increasing the poor’s access to institutions and lifting discriminatory practices. From a practical standpoint, this approach emphasizes the importance of increasing access and opportunities. Another, rather distinct approach to thinking about the poor is offered by the culture of poverty theory^42,43^, which contends that people in a state of scarcity have immutable, “deviant” preferences that would dampen the effectiveness of such policies.

In documenting the powerful effects of agency on behavior, our findings point to a different approach: structuring programs to provide individuals with greater agency. In addition to any instrumental or intrinsic benefits of agency, our results suggest such programs could lead to more farsighted behavior. This shift could increase the likelihood that program participants engage in behaviors associated with immediate costs and larger, delayed rewards, such as investing in education and saving for a rainy day. Note that because, in our studies, increased agency shifted behavior even when the agency was not exercised, agency-based interventions have the potential to cultivate more patient choices^44^ while keeping program costs relatively low.

## Methods

### Study 1

This experiment was approved by the Carnegie Mellon University Institutional Review Board. It complied with all relevant ethical regulations and involved informed consent. All data were collected using the Qualtrics survey platform.

Participants were recruited from Amazon Mechanical Turk (*N* = 217) and randomly assigned to one of three conditions that manipulated the level of scarcity and agency: Scarcity–Agency (hereafter Agency), Scarcity–No Agency (hereafter No Agency), and a condition with no scarcity (hereafter Control). In the first part of the study, all participants responded to a set of 15 true–false cognitive aptitude questions adapted from the Wonderlic Cognitive Ability Test (e.g., “A farmer had 17 sheep. All but 9 died. There are 36 feet on the remaining sheep.”)

We manipulated resource scarcity by varying the amount of time participants had to answer the questions. This manipulation builds on prior work in the scarcity literature that used time scarcity as an analog of financial or resource scarcity^30^. Participants in both scarcity conditions faced time scarcity: they had 10 seconds to answer each question, at the end of which the screen automatically advanced to the next question. Participants in the Control condition (*N* = 72) did not face scarcity—they had unlimited time to answer the questions. All participants were paid a base payment and a bonus for each correct answer.

Next, participants answered a second set of 15 true–false cognitive aptitude questions under a different incentive structure. Participants were informed that once they were done, a number between 1 and 15 would be randomly drawn to serve as a threshold. Participants’ whose number of correct answers met or exceeded this threshold would receive a bonus, which would double their payment; otherwise, they would receive no bonus. For this second task, participants in the scarcity conditions experienced increased scarcity: they now had only six (rather than 10) seconds to answer each question. See Supplementary Fig. 3 for a description of how much time scarcity this manipulation induced. Participants in the Control condition, again, faced no time scarcity.

To manipulate agency over scarcity, participants in the No Agency condition (*N* = 76) were not given the option to increase the time per question in the second set of questions, whereas those in the Agency condition (*N* = 69) were given that option. Specifically, we provided those in the Agency condition with the option to add four seconds per question. Participants were informed about this option before they started the second set of questions. They were allowed to exercise their agency either before the task, or at any point while answering the questions, by checking a box on the screen. Critically, alleviating the scarcity came at a cost: participants were told that by checking the box, they would forfeit a substantial portion (80%) of their base payment. We added this cost to discourage participants from exercising their agency, ensuring that actual scarcity experienced by those in the Agency and No Agency conditions would be the same. Those who did choose to gain more time (*N* = 4) were routed out of the study at the end of the cognitive aptitude task. They did not complete the dependent variable tasks and cannot be included in our main analyses.

After completing the second set of questions, participants were asked to indicate their sense of agency and the extent to which they were angry, sad, and happy (on a scale from 1 = “not at all” to 5 = “very”). To minimize the potential for income effects, we did not inform participants about their earnings for the second set of cognitive aptitude questions until the end of the experiment.

Next, we elicited participants’ time preferences by asking them to allocate 100 experimental tokens across two dates^45^. The value of a token allocated to later dates was 50% higher than the value of a token allocated to an earlier date, such that patience was rewarded with a larger payment. Participants made two separate allocation decisions. In the first, they allocated 100 tokens between “today” and “one week from today.” In the second, they allocated a separate set of 100 tokens between “one week from today” and “two weeks from today.” To make the choices consequential, we informed participants that one of their two allocation decisions would be randomly chosen, and the corresponding amounts would be paid as a bonus on the date associated with the choice. Patience was captured by the number of tokens the participant allocated to the later date, representing her willingness to wait for a larger, later reward over a smaller, sooner one. Immediately following the second allocation task, participants were given a comprehension check to test their understanding of the task.

Participants then answered a set of four hypothetical questions that elicited a binary preference between a safer and a riskier option^46^. Finally, to check whether our scarcity manipulation was effective, we asked participants to indicate the extent to which they felt they had enough time to answer the cognitive aptitude questions (on a scale from 1 = “I did not have enough time” to 7 = “I had too much time”). We report all data exclusions, all manipulations, and all measures. See Supplementary Method 2 for details on measures and instructions.

### Study 2

This experiment was approved by the University of California, San Diego (UCSD) Institutional Review Board. It complied with all relevant ethical regulations and involved informed consent. All data were collected using the Qualtrics survey platform. Undergraduates taking classes at the Rady School of Management (*N* = 115) participated in a laboratory experiment in exchange for class credit. This context allows us to test whether our results generalize to a different participant population than was used in Study 1^47,48^.

In operationalizing an adverse state, we built on a classic helplessness paradigm^49^ that exposes participants to environmental stressors in the form of aversive, unpredictable noise. All participants were told they would be asked to solve 30 anagrams in five minutes while listening to a loud, jarring noise (3000 Hz, 90 dB tone at random intervals) through a set of headphones. We provided them with examples of anagrams (e.g., TIGF is an anagram for GIFT) and informed them that at the end of the study, we would randomly choose one in 10 participants to receive a $20 bonus.

Participants were instructed to put on the headphones that were connected to their computer and were randomized into either the Agency (*N* = 61) or No Agency (*N* = 54) condition. We informed participants in the No Agency condition that removing the headphones would disqualify them from the study, and thus from the possibility of receiving the bonus payment. In contrast, we told participants in the Agency condition that they had the option to remove the headphones at any time, but that doing so would cost them 50% of the potential bonus payment. Similar to Study 1, we added this cost to discourage participants from exercising their agency, ensuring participants in both conditions experienced the same level of jarring noise. Participants completed four practice anagrams without noise before beginning the real anagram task.

Immediately after completing the main task of 30 anagrams, we elicited participants’ levels of patience by asking them to make a series of 27 hypothetical choices between a smaller, sooner reward and a larger, later reward (e.g., “Would you prefer $14 today or $25 19 days from now?”). The smaller (sooner) amount, the larger (later) amount, and the delay between the payouts varied across questions, allowing us to estimate patience using both nonparametric and parametric techniques^35^. See Supplementary Method 4 for detailed measures and instructions.

### Reporting summary

Further information on research design is available in the Nature Research Reporting Summary linked to this article.

## Supplementary information

Supplementary Information

Reporting Summary

Description of Additional Supplementary Files

Supplementary Data 1

Supplementary Data 2

Supplementary Data 3

Supplementary Data 4

Supplementary Data 5

Supplementary Software 1

Supplementary Software 2

Supplementary Software 3

Supplementary Software 4

Supplementary Software 5

## Data Availability

The authors declare that all data generated for this paper are included in the Supplementary Information files (Supplementary Data 1 through Supplementary Data 5). The studies described in the paper and Supplementary Notes were collectively run between 2012 and 2018. The data for the World Values Survey analysis is publicly available at http://www.worldvaluessurvey.org/WVSDocumentationWV6.jsp.

## Code availability

The authors declare that all analysis used to generate the conclusions in this paper are included in the Supplementary Information files (Supplementary Code 1 through Supplementary Code 5). All data were analyzed using Stata 16.
