# Supplementary material for: The impact of agency on time and risk preferences
Source: Nat Commun. 2020 May 29;11:2665. doi: 10.1038/s41467-020-16440-0 (PMC7260199; doi:10.1038/s41467-020-16440-0)
Supplement: Supplementary file 1 — Supplementary Information [file 41467_2020_16440_MOESM1_ESM.pdf]

Supplementary Information

The Impact of Agency on Time and Risk Preferences

Ayelet Gneezy, Alex Imas, & Ania Jaroszewicz

Supplementary Information content in this PDF:

Supplementary Methods 1 to 5
Supplementary Notes 1 to 4
Supplementary Figures 1 to 3
Supplementary Tables 1 to 6
Supplementary References

Other Supplementary Information content:

Supplementary Datasets 1 to 5
Supplementary Code 1 to 5

Supplementary Method 1 – 2010-2014 World Values Survey Questions

Question 55: Some people feel they have completely free choice and control over their lives, while other people feel that what they do has no real effect on what happens to them. Please use this scale where 1 means 'none at all' and 10 means 'a great deal' to indicate how much freedom of choice and control you feel you have over the way your life turns out.

Question 229: Are you employed now or not? If yes, about how many hours a week? If more than one job: only for the main job:

- Full time employee (30 hours a week or more)
- Part time employee (less than 30 hours a week)
- Self employed
- Retired/pensioned
- Housewife not otherwise employed
- Student
- Unemployed
- Other (write in)

Question 237: During the past year, did your family:

- Save money
- Just get by
- Spent some savings *[sic]*
- Spent savings and borrowed money *[sic]*

Supplementary Method 2 – Study 1 Materials

(Manipulations—orange font pertains to Control group, dark blue font pertains to groups experiencing scarcity [No Agency and Agency], red font pertains to Agency group.)

Instructions

You will be given 15 True-False questions to answer. **Your payment will depend partly on your answers to these questions.**

You will begin with 50 cents. For each question you answer correctly, you will be paid 2 additional cents. You will earn no additional money if you answer incorrectly or don't answer the question at all.

[**You will have as much time as you need to answer each question.** After you answer each question, you will see whether you got the answer right or wrong.**]** **[You will have 10 seconds to answer each question.** After the 10 seconds are over, you will see whether you got the answer right or wrong. The screen will then automatically advance to the next question.]

The questions will begin when you click on the ">>" button below.

(Next page. Each of the 15 questions below had the options "True" and "False" and appeared on its own page for either 10 seconds [for the groups experiencing scarcity, i.e. No Agency and Agency] or for an unlimited period of time [for the Control group]. A large green checkmark appeared after correct answers were chosen, while a large red X appeared after an incorrect answer, or no answer, was chosen. The feedback remained on the screen for two seconds before the screen automatically advanced to the next question.)

There are ten letters in the word **COMMITMENT.**

Emma drives 10 miles to work and back every work day, and 2 miles to the gym and back on each day of the weekend. She drives for 54 miles every week.

John and Mary have bicycles. Their little sister Elizabeth has a tricycle. If they all go out for a ride, they have six wheels altogether.

The letters in the word "Chimney" are in alphabetical order.

The sister of your brother's father is your aunt.

There are only two vowels in each of the following names: Emma, Adam, Emil, Nathan, Eli.

The combination of two of the numbers: 1, 3, 4, 7, 8 is equal to 13.

Roy smokes a pack of cigarettes every three days. He smokes seven packs every three weeks.

A farmer had 17 sheep. All but 9 died. There are 36 feet on the remaining sheep.

The word **FEEL** can be formed with exactly 12 match sticks without bending or breaking any of them.

A number of children are standing a circle. They are evenly spaced and the 8th child is directly facing the 16th child. There are 17 children in the circle.

Lucy made three steps to the North, after that she took a step to the East and three more steps to the South. She is now at her starting position.

Each child in a family has at least 5 brothers and 3 sisters. The smallest number of children the family might have is 10.

The word **VILE** can be written using four of the letters of the word **VIOLATES**.

If you write down all the numbers from 1 to 25, every two numbers following one another will add up to an odd number.

(Next page.)

You will now be presented with 15 more questions. Once you are finished with the questions, we will randomly choose a number between 1 and 15. If the number of questions you answer correctly **is at least as big as that number, you will receive 50 cents.** Otherwise, you will receive no additional payment. **The more questions you answer correctly, the higher your chances of being paid.**

[You will have as much time as you need to answer each question.] **[**In addition, **you will only have 6 seconds instead of 10 seconds to answer each question.]**

(Next page.)

You have been given the control to gain an additional 4 seconds per question in exchange for paying a portion of your baseline fee (40 cents). If you choose to exercise this option, you will have 10 seconds per question. Instead of receiving 50 cents as your baseline fee, you will receive 10 cents and whatever you earn for answering the questions.

To pay 40 cents for an additional 4 seconds per question, check the box below. Otherwise, just click the ">>" button at the bottom of the screen to begin.

Note that if you choose not to pay now, you will continue having the control to do so after starting the questions, as well.

The questions will begin when you click on the ">>" button below.

☐**Yes, I would like to pay 40 cents in exchange for an additional 4 seconds per question**

(Next page. Each of the 15 questions below had the options "True" and "False" and appeared on its own page. Those in the conditions experiencing scarcity [No Agency and Agency] saw each question for only six seconds, while those in the Control condition saw each question for as long as they wanted. Those in the Agency group also saw the option in red font. If they clicked the box, all questions thereafter remained visible for four seconds longer and the sentence in red disappeared. As before, a green checkmark appeared after correct answers, and a red X appeared after incorrect or blank answers.)

13 minutes after 5 o'clock is exactly 47 minutes before 6 o'clock.

☐I would like to pay 40 cents in exchange for an additional 4 seconds per question for all questions that follow.

The word **AMITY** is written by using the first letters of the sentence "A mouse in the yard".

☐I would like to pay 40 cents in exchange for an additional 4 seconds per question for all questions that follow.

If Peter looks at a mirror and touches his left ear, his mirror image would touch his right ear.

☐I would like to pay 40 cents in exchange for an additional 4 seconds per question for all questions that follow.

Using exactly three colors you can paint the sides of a cube so that sides of the same color will never touch.

☐I would like to pay 40 cents in exchange for an additional 4 seconds per question for all questions that follow.

The third vowel in this sentence is "o".

☐I would like to pay 40 cents in exchange for an additional 4 seconds per question for all questions that follow.

Seven chickens and two cats have twenty-two legs among them.

☐I would like to pay 40 cents in exchange for an additional 4 seconds per question for all questions that follow.

There are four letters between the letter K and the letter P in the alphabet.

☐I would like to pay 40 cents in exchange for an additional 4 seconds per question for all questions that follow.

If the word ERA is written below the word ANT and the word RAT is written below ERA, then the word ART will form diagonally.

☐I would like to pay 40 cents in exchange for an additional 4 seconds per question for all questions that follow.

.55 hours added to half an hour is exactly 85 minutes.

☐I would like to pay 40 cents in exchange for an additional 4 seconds per question for all questions that follow.

If Tuesday is the second day of the month, then the first Sunday will be the sixth day of the month.

☐I would like to pay 40 cents in exchange for an additional 4 seconds per question for all questions that follow.

62 is the next logical number in the sequence: 2,6,14,30

☐I would like to pay 40 cents in exchange for an additional 4 seconds per question for all questions that follow.

Statement 1: The boy plays baseball. Statement 2: All baseball players wear hats. Statement 3: The boy wears a hat. Assume the first 2 statements are true. The final one is:

☐I would like to pay 40 cents in exchange for an additional 4 seconds per question for all questions that follow.

A train travels 20 feet in 1/5 second. At this same speed, the train will travel 60 feet in three seconds.

☐I would like to pay 40 cents in exchange for an additional 4 seconds per question for all questions that follow.

A boy is 17 years old and his sister is twice as old. When the boy is 23 years old, his sister is 40 years old.

☐I would like to pay 40 cents in exchange for an additional 4 seconds per question for all questions that follow.

If you rearrange the letters **ANICH** you will get the name of a country.

☐I would like to pay 40 cents in exchange for an additional 4 seconds per question for all questions that follow.

(Next page. Questions were answered on a scale from 1 [Not at all] to 5 [Very]. Participants who chose to gain more time were sent straight to the demographic questions.)

Please indicate to what extent you feel…

Angry
Happy
Empowered

Sad

(Next page.)

Instructions

In the next section, you will be asked to make two decisions about how to divide a set of 100 tokens between two dates. **Your earnings will depend on these choices.**

The tokens you allocate to the later date will always be worth more money than the tokens you allocate to the earlier date. This process is best described by an example.

The decision on the screenshot below shows the choice to allocate 100 tokens between two dates: today, and tomorrow. In this decision, each token you allocate to today is worth $0.10, while each token you allocate to tomorrow is worth $0.20.

So, if you allocate 80 tokens to today and 20 tokens to tomorrow, the value of the tokens is 80 x $0.10 = $8.00 today, and 20 x $0.20 = $4.00 tomorrow.

On the other hand, if you allocate 20 tokens to today and 80 tokens to tomorrow, the value of the tokens is 20 x $0.10 = $2.00 today, and 80 x $.20 = $16.00 tomorrow.

Please remember that your earnings will depend on your responses to these questions. One of your choices will be randomly chosen and on the specified date, you will be given a bonus equal to 1/100 of the value you allocate to that date.

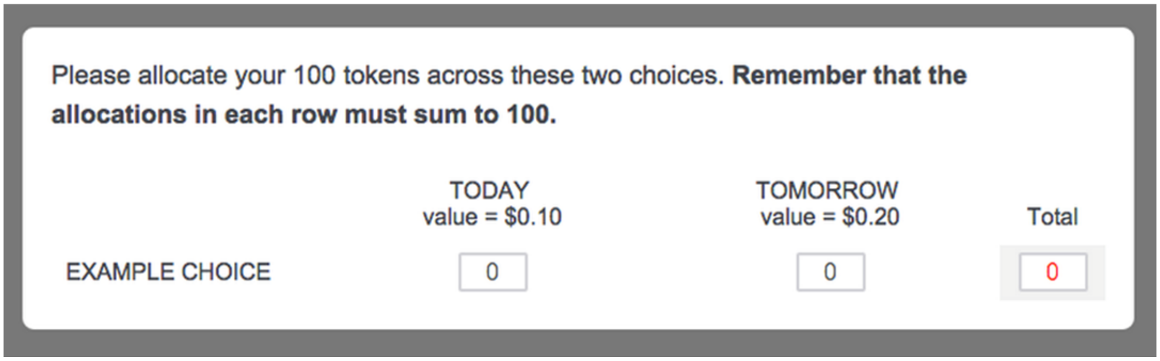

(Next page. Total automatically calculated. Participants could not proceed if the total did not equal 100.)

Please allocate your 100 tokens across these two choices. **Remember that the allocations in each row must sum to 100.**

	TODAY value = $0.20	IN ONE WEEK value = $0.30	Total
Choice 1	0	0	0

Please allocate your 100 tokens across these two choices. **Remember that the allocations in each row must sum to 100.**

	IN ONE WEEK value = $0.20	IN TWO WEEKS value = $0.30	Total
Choice 2	0	0	0

(Next page.)

Suppose the values of the tokens are $0.20 for today and $0.30 one week from now. If you allocate 50 tokens to today and 50 tokens to one week from now, what is the value of the tokens (before we divide them by 100)?

- o $10 today and $20 in one week
- o $10 today and $15 in one week
- o $20 today and $30 in one week

(Next page.)

For the following sets of gambles, please choose the gamble that you would prefer.

Please choose between the following gambles:

$2,500 with probability .33<br>$2,400 with probability .66<br>$0 with probability .01	$2,400 with certainty
O	O

Please choose between the following gambles:

$2,500 with probability .33<br>$0 with probability .67	$2,400 with probability .34<br>$0 with probability .66
O	O

Please choose between the following gambles:

$4,000 with probability .80<br>$0 with probability .20	$3,000 with certainty
O	O

Please choose between the following gambles:

$4,000 with probability .20<br>$0 with probability .80	$3,000 with probability .25<br>$0 with probability .75
O	O

(Next page. 7-point scale ranging from "I did not have enough time" to "I had too much time".)

Did you have enough time to answer the timed true-false questions earlier in the survey?

(Next page.)

Gender

- Male
- Female

Race

- White
- Black or African American
- Hispanic (non-white)
- Asian
- Other / mix
- Prefer not to answer

Are you fluent in English?

- No
- Yes

Are you a student?

- No
- Yes

What is your annual income, after taxes and deductions?

- 0 - $10,000
- $10,000 - $20,000
- $20,000 - $40,000
- $40,000 - $60,000
- $60,000 - $80,000
- $80,000 - $100,000
- $100,000 - $150,000
- over $150,000

(Next page. Text box.)

Finally, we'd like to ask you on your thoughts about the experiment. Did anything seem unusual or unclear? Did you run into any difficulties?

(Next page. Font in purple appeared only for individuals who did not choose to gain more time. Time periods in curly brackets dependent on which time preferences question was chosen to be realized. Font in green appeared only for individuals who chose to gain more time.)

Thank you for your time! Your responses have been recorded and the experiment is now over.

You began with 50 cents. In the first set of questions, you answered [#] questions correctly, where each question was worth 2 cents. In the second set of questions, you answered [#] questions correctly, for a total of [$]. You also earned a bonus payment of [$] today {in one week} and [$] in one week {in two weeks}. You chose to pay 40 cents of your baseline fee to gain an additional 4 seconds of time per question.

Thus, altogether, your earnings are: [$].

Please proceed to the next screen to receive your Mechanical Turk completion code.

Supplementary Method 3 – Study 1b Materials

(Instructions are identical to those of Study 1 through the end of the second set of cognitive aptitude questions. Participants who chose to exercise their agency and gain more time did not complete the demand for agency measure. Instead, they were routed to the demographics section.)

We will now flip a fair coin. You have the opportunity to potentially earn an additional bonus based on the outcome of the coin flip. You will have a "winning side" -- either Heads or Tails. If the outcome of the coin flip matches your "winning side," you will win an additional $1.00 bonus. If the coin comes up on the other side, you will lose and not get an additional bonus.

You have the opportunity to choose your "winning side." You will first state the *highest amount you are willing to pay to choose your "winning side"* (from $0.00 to $0.50). After you state this amount, we will choose a random number from $0.00 to $0.50. If your willingness to pay is lower than the random number, we will choose your side for you. If your willingness to pay is equal to or higher than the random number, the random number will be deducted from the bonus (should you win the coin flip) and you will get to choose your "winning side." An amount will only be deducted from your bonus if you win the coin flip.

So, the more you are willing to pay, the more likely it is that you will be able to choose your "winning side" of the coin.

How much are you willing to pay to choose your "winning side" of the coin? *(Sliding scale from $0.00 to $0.50 with a label of "Your willingness to pay.")*

(Next page. Scale labeled with 1=Very slightly or not at all, 2=A little, 3=Moderately, 4=Quite a bit, 5=Extremely. Items were presented in random order.)

Please indicate to what extent you currently feel...

- Angry
- Sad
- Upset

(Next page.)

To what extent do you agree with this statement? "During this experiment, I trusted that the experimenter would do what the instructions said they would do." *(1=Do not at all agree, 2=Agree a little, 3=Agree a moderate amount, 4=Agree a lot, 5=Agree a great deal.)*

Did you have enough time to answer the true-false questions earlier in the survey? *(7 point scale; 1=I did not have enough time, 4=I had the right amount of time, 7=I had too much time.)*

(Next page.)

Gender
- Male
- Female
- Non-conforming

Race
- White
- Black or African American
- Hispanic (non-white)
- Asian
- Other / mix
- Prefer not to answer

Are you fluent in English?
- No
- Yes

Are you a student?
- No
- Yes

What is your annual income, after taxes and deductions?
- 0 - $10,000
- $10,000 - $20,000
- $20,000 - $40,000
- $40,000 - $60,000
- $60,000 - $80,000
- $80,000 - $100,000
- $100,000 - $150,000
- over $150,000

(Next page. For participants whose willingness to pay was lower than the randomly chosen number, half were randomized to have their "winning side" be Heads, and half were randomized to have their "winning side" be Tails.)

Your willingness to pay was lower than the random number. Your "winning side" will be Heads [Tails].

(Next page. For participants whose willingness to pay was higher than the randomly chosen number:)

Your willingness to pay was higher than the random number. **Please pick your "winning side" of the coin:**

- Heads
- Tails

(Next page. Image of a US quarter on the appropriate side was pictured below text. Each participant was randomized to see either the Heads or Tails outcome.)

The outcome of the coin flip was Heads [Tails].

(Next page.)

Thank you for your time! Your responses have been recorded and the experiment is now over.

You began with 50 cents. In the first set of questions, you answered [#] questions correctly, where each question was worth 2 cents. In the second set of questions, you answered [#] questions correctly, for a total of [$]. You earned [$] during the coin flip game. You will receive these additional earnings as a bonus in the next few days.

Please proceed to the next screen to receive your Mechanical Turk completion code.

Supplementary Method 4 – Study 2 Materials

Instructions *(for **No Agency** condition)*
Welcome to our experiment. To thank you for your participation, we will randomly choose 1 in 10 participants to receive $20. If you are chosen to be paid, we will notify you by email and pay you next week.
Please put on the headphones located at your station.
The aim of the first part of the experiment is to examine how environmental factors impact performance in simple word tasks. You will be asked to solve a series of anagrams. Anagrams are words with the letters jumbled.
For example:
TIGF is an anagram for GIFT
LERU is an anagram for RULE
UMOSE is an anagram for MOUSE
You will be given 30 anagrams, and asked to solve as many as possible in 5 minutes. The solution to each anagram is a very well known word (not a person's name), and each anagram has only one such solution.
Importantly, while solving the anagrams you may be exposed to noise through your headphones. Note that removal of the headphones at any point will disqualify you from the study, which means you will not be eligible to be paid.

Instructions *(for **Agency** condition)*
Welcome to our experiment. To thank you for your participation, we will randomly choose 1 in 10 participants to receive $20. If you are chosen to be paid, we will notify you by email and pay you next week.
Please put on the headphones located at your station.
The aim of the first part of the experiment is to examine how environmental factors impact performance in simple word tasks. You will be asked to solve a series of anagrams. Anagrams are words with the letters jumbled.
For example:
TIGF is an anagram for GIFT
LERU is an anagram for RULE
UMOSE is an anagram for MOUSE
You will be given 30 anagrams, and asked to solve as many as possible in 5 minutes. The solution to each anagram is a very well known word (not a person's name), and each anagram has only one such solution.
Importantly, while solving the anagrams you may be exposed to noise through your headphones. Note that you can choose to remove your headphones at any point during the experiment at a cost of 50% of your payment should you be chosen to get paid. That is, if you choose to remove your headphones, half of your final payment will be deducted if you are chosen to be paid.

(Next page.)

Please proceed to the next page to practice solving anagrams while being exposed to noise.

(Next page.)

WHYROT
LOMUVE
MILTEY
CLAUHN

(Next page.)

Please inform the experimenter NOW if you have any questions.
Otherwise, please proceed to the next page and begin solving the anagrams.

(Next page.)

Solve as many anagrams as you can. Enter your solution into the Text Box below each word. You have 5 minutes.

(While solving the anagram task, participants listened to a loud, jarring noise [3,000 Hz, 90dB tone at random intervals]. Recordings of the noise are available at https://sites.google.com/site/alexoimas/Unpredictable_3.mp3.)

MEECHS
INGALC
IBINIK
CERAPH
PIRAMI
CLINEP
NERCRO
AHVEBE
PRINGY
ROTHEY
TACTIN
URAUBE
WHOSAD
ASHRIP
JEERTS
YOUTCH
SPOMIE
DANINL
ENGLOB
POWNEA
HEHRST
MODDEO
CIRPAY
GIRONI
DILQUI

TORFOG
GOAUNT
RELPHE
DORPAY
LENZOZ

(Next page.)

You will now be presented with a series of 27 choices. Please take the choices seriously, and make each decision as though it will actually be carried out. In each case, you will be presented with a choice between an immediate reward and a delayed reward. Please choose the one you would prefer.

Would you prefer $54 dollars today or $55 dollars 117 days from now?
- $54 dollars today
- $55 dollars 117 days from now

Would you prefer $55 dollars today or $75 dollars 61 days from now?
- $55 dollars today
- $75 dollars 61 days from now

Would you prefer $19 dollars today or $25 dollars 53 days from now?
- $19 dollars today
- $25 dollars 53 days from now

Would you prefer $31 dollars today or $85 dollars 7 days from now?
- $31 dollars today
- $85 dollars 7 days from now

Would you prefer $14 dollars today or $25 dollars 19 days from now?
- $14 dollars today
- $25 dollars 19 days from now

Would you prefer $47 dollars today or $50 dollars 160 days from now?
- $47 dollars today
- $50 dollars 160 days from now

Would you prefer $15 dollars today or $35 dollars 13 days from now?
- $15 dollars today
- $35 dollars 13 days from now

Would you prefer $25 dollars today or $60 dollars 14 days from now?
- $25 dollars today
- $60 dollars 14 days from now

Would you prefer $78 dollars today or $80 dollars 162 days from now?

- $78 dollars today
- $80 dollars 162 days from now

Would you prefer $40 dollars today or $55 dollars 62 days from now?
- $40 dollars today
- $55 dollars 62 days from now

Would you prefer $11 dollars today or $30 dollars 7 days from now?
- $11 dollars today
- $30 dollars 7 days from now

Would you prefer $67 dollars today or $75 dollars 119 days from now?
- $67 dollars today
- $75 dollars 119 days from now

Would you prefer $34 dollars today or $35 dollars 186 days from now?
- $34 dollars today
- $35 dollars 186 days from now

Would you prefer $27 dollars today or $50 dollars 21 days from now?
- $27 dollars today
- $50 dollars 21 days from now

Would you prefer $69 dollars today or $85 dollars 91 days from now?
- $69 dollars today
- $85 dollars 91 days from now

Would you prefer $49 dollars today or $60 dollars 89 days from now?
- $49 dollars today
- $60 dollars 89 days from now

Would you prefer $80 dollars today or $85 dollars 157 days from now?
- $80 dollars today
- $85 dollars 157 days from now

Would you prefer $24 dollars today or $35 dollars 29 days from now?
- $24 dollars today
- $35 dollars 29 days from now

Would you prefer $33 dollars today or $80 dollars 14 days from now?
- $33 dollars today
- $80 dollars 14 days from now

Would you prefer $28 dollars today or $30 dollars 179 days from now?
- $28 dollars today
- $30 dollars 179 days from now

Would you prefer $34 dollars today or $50 dollars 30 days from now?

- $34 dollars today
- $50 dollars 30 days from now

Would you prefer $25 dollars today or $30 dollars 80 days from now?

- $25 dollars today
- $30 dollars 80 days from now

Would you prefer $41 dollars today or $75 dollars 20 days from now?

- $41 dollars today
- $75 dollars 20 days from now

Would you prefer $54 dollars today or $60 dollars 111 days from now?

- $54 dollars today
- $60 dollars 111 days from now

Would you prefer $54 dollars today or $80 dollars 30 days from now?

- $54 dollars today
- $80 dollars 30 days from now

Would you prefer $22 dollars today or $25 dollars 136 days from now?

- $22 dollars today
- $25 dollars 136 days from now

Would you prefer $20 dollars today or $55 dollars 7 days from now?

- $20 dollars today
- $55 dollars 7 days from now

Supplementary Method 5 – Study 2b Description of Method

We assigned participants to isolated computer stations and instructed them to put on a pair of headphones. We randomly assigned participants to either an Agency or No Agency condition.

As in Study 2, participants in both conditions heard a 3,000 Hz, 90 dB tone at random intervals (somewhat similar to a powerful vacuum cleaner). We instructed participants in the No Agency condition to keep their headphones on for the duration of the experiment or be disqualified and forfeit all potential earnings. Instructions in the Agency condition instead read: "Note that you can choose to remove your headphones at any point during the experiment at a fixed cost of 50 tokens. That is, if you choose to remove your headphones, 50 tokens will be deducted from your final payment."

Next, we informed participants they were matched with another individual against whom they would compete in solving as many anagrams as possible within five minutes. The winner would receive 200 tokens, while the loser would receive 100 tokens (10 tokens = $1). We further informed participants that either they, or their opponent, would be randomly given the opportunity to impose an offensive and distracting noise on the other player during the task. In practice, participants engaged with a programmed simulation of the interaction, allowing us to maintain a uniform level of adversity across conditions.

Prior to the anagram competition, participants completed two 15-second practice sessions consisting of five anagrams each—one with noise and one without. The actual anagram competition began after they were informed their opponent had been chosen to have the option of activating the aversive noise: participants in both conditions had five minutes to solve as many anagrams as they could.

After the anagrams task, participants were told that they could bid against their opponent for the option to choose the winning outcome of a computer-generated coin toss (heads or tails) in which the winner would be awarded an additional 100 tokens. We also informed participants the bid amount would only be deducted from the winner's earnings. Bids could range from 0 to 50 tokens.

Supplementary Note 1 – Robustness Checks on Study 1

As an additional robustness check, we analyze our data from Study 1 while controlling for participants' responses to the time preference comprehension check question. In the comprehension check, participants were told about particular token values for "today" and "one week from today" and asked to identify the total token value at each date, conditional on allocating the tokens evenly across the two dates. Participants were provided with three response options to the question: one that was fully correct, one that was partially correct, and one that was fully incorrect. We find that the majority (78%) of participants answered the time preferences comprehension check question fully correctly, significantly greater than the benchmark for random choice ($p<0.00005$ for each of the three conditions).

We can code our measure of comprehension in several ways. One method is to code a binary variable corresponding to 0 if the participant chose the fully incorrect option and 1 otherwise. Alternatively, one could create a categorical variable that is equal to 0 for a participant who chose the fully incorrect response, 1 if she chose the partially incorrect option, and 2 if she chose the fully correct option. A third method is to create a binary variable corresponding to 0 if the participant did not choose the fully correct option and 1 otherwise. Irrespective of which coding method we adopt, the results show that including this measure in our analyses does not substantially change our results.

An OLS regression with a binary indicator for the No Agency group shows the coefficient on the No Agency treatment indicator variable remains around 24-30% of one standard deviation of the mean tokens allocated to the earlier date, regardless of the specification (robust SE clustered at the participant ID level, comparing only the No Agency to the Agency group). Rerunning the analysis when including all three treatment groups yields similar results: the coefficient of the No Agency group remains around 29%-32% of one standard deviation of the mean tokens allocated to the earlier dates, depending on the specification.

As an additional robustness check, we also calculate Lee bounds on our treatment effects[1]. This measure allows us to address the potential selection concern by offering a more conservative estimate of our results. We test the difference between the Agency and No Agency groups while including a median-split covariate of score for the first set of cognitive aptitude questions. Our analyses reveal that the 95% confidence interval for the treatment effect does not include 0 for either the patience (0.32, 16.91) or risk tolerance (0.04, 0.25) measures.

Finally, we reanalyze our risk results using a subset of the risk preferences questions. Of the four risk preferences questions included, two measured preferences for a certain outcome over an uncertain one, while two measured preferences between two uncertain outcomes. To test whether our results are driven by general risk aversion or by a specific preference for complete certainty, we rerun our analyses using participants' responses to only the two questions measuring preferences between two uncertain outcomes. We find that our conclusions remain unchanged. Participants in the No Agency group continue to be significantly more risk averse than those in the Agency group (two-tailed pairwise t-test, $t(139)=2.568$, $p=0.011$), while there continue to be no differences for either of the other two pairwise comparisons (all $ps\geq0.188$).

Supplementary Note 2 – Variations on Study 1

In addition to the studies reported in the main text, we ran a variation on Study 1 where participants were randomized into one of four conditions that manipulated both time scarcity (the adverse state) and agency, leading to a 2 (Less Scarcity vs. Scarcity) x 2 (No Agency vs. Agency) between-subjects design. Unlike in the Study 1 described in the main body of the paper, Less Scarcity participants did not have unlimited time to answer the cognitive aptitude questions—instead, they had 10 seconds per question in both the first and second set of questions (i.e., they faced less scarcity than the participants in the two scarcity groups for the second set of cognitive aptitude questions). Second, these studies had an additional treatment, Less Scarcity – Agency: participants in this group, who already had 10 seconds to answer each question, were granted the option to gain an additional four seconds per question in the second set of questions for the cost of 50% of the baseline compensation. Third, participants did not receive feedback on their performance for either set of cognitive aptitude questions until the end of the task. Finally, the payoff structure was slightly different: the baseline fee was lower (40 cents instead of 50 cents), as was the bonus for having more correct answers than the threshold in the second set of questions (25 cents instead of 50 cents). In addition, the cost of gaining more time was also lower (50% of the baseline fee, rather than 80%). Similar to the procedure of Study 1, individuals who chose to gain more time were routed out of the study and their time preferences data were not collected, ensuring all Scarcity participants included faced the same level of adversity.

We ran this study twice. In the first iteration (Study #1, N=178), 20 participants chose to gain more time. Of the remaining participants, those in the Scarcity – No Agency group allocated more tokens to the earlier date—i.e., they were more impatient than Scarcity – Agency group participants (p=0.03), as well as both No Scarcity group participants (both ps<0.01). In the second iteration (Study #2, N=182), seven participants chose to gain more time. Here, scarcity did not influence time preferences, and thus endowing participants with agency had a null effect (all ps>0.10).

As a robustness check of the results, we pool our results across these two iterations and Study 1 of the paper, and compare participants' choices in the Scarcity – No Agency and Scarcity – Agency conditions (labeled as No Agency and Agency in the body of the text). Although marginally significant, we find that participants in the Scarcity – No Agency group were more impatient than those in the Scarcity – Agency group, consistent with our prediction (see Supplementary Tables 3 and 4).

Supplementary Note 3 – Study 1 Manipulation Check Study (Study 1b)

We ran a follow up study, Study 1b, to test whether our manipulation was indeed affecting agency and to ensure that the observed differences in Study 1 were not driven by changes in other measures. Prior work has demonstrated that within-participant self-report measures are not neutral – they are sensitive to context and potentially affect responses on the tasks that follow[2]. For this reason, we conducted a separate study to demonstrate that our manipulation shifted participants' sense of agency by using a behavioral measure that captured subsequent demand for agency. All materials for the study, including the data and analysis code, are included in the Supplementary Information files. The study was approved by the Carnegie Mellon University IRB. It complied with all relevant ethical regulations and involved informed consent.

We randomly assigned participants (N=221) into one of the same three conditions used in the Study 1 described in the main body of the paper: No Agency (N=79), Agency (N=77), and Control (N=65). The first part of the study was identical to that of Study 1 up to the part where time and risk preferences were elicited. Then, instead of eliciting these preferences, we measured demand for agency using a task based on those used in classic agency studies[3]. Participants were informed that a computer would flip a virtual fair coin. If the coin flip matched their "winning side" (Heads or Tails), they would win a $1.00 bonus; if it did not, they would win nothing. We then elicited an incentivized measure of participants' maximum bid to choose their preferred side of the coin. The measure was incentivized in the following manner: if the participant's bid exceeded a randomly drawn number (up to $0.50), she would choose her "winning side." If she won the coin flip, the random number would be deducted from her earnings; if she did not win the coin flip, nothing would be deducted from her earnings. If the participant's bid was less than the randomly drawn number, the "winning side" would be assigned to her and nothing would be deducted from her earnings regardless of whether she won the coin flip or not.

Prior work on the illusion of control[3] has demonstrated that people often perceive they can exert control over chance events such as coin flips. As such, we predicted that although choosing the "winning side" has absolutely no effect on the coin flip outcome or the participant's chance of winning, people would have a positive willingness to pay to choose their "winning side." Importantly, we also predicted those with a lack of agency (No Agency condition) would have a greater subsequent demand for agency over their environment than those who already had a greater sense of agency (Agency condition), and thus have a higher willingness to pay to exert control over their environment and choose their "winning side" of the coin flip. The reasoning is as follows: if people have a preference to have more agency rather than less[4,5,6,7], and demand for agency can be modeled using a well-behaved utility function (see prior work[8] for evidence), then compared to people with high agency, those with low agency should be willing to pay more to gain additional agency.

Thirteen participants chose to gain more time. Consistent with our predictions, bids in the No Agency condition ($M_{NoAgency}$=\$0.18) were significantly higher than those in the Agency condition (M_{Agency}=\$0.11; two-tailed pairwise t-test, $t(141)=2.177$, $p=0.031$). We further find no significant differences between the No Agency and Control conditions ($M_{Control}$=\$0.15; two-tailed pairwise t-test, $t(142)=0.712$, $p=0.459$), nor between the Agency and Control conditions (two-tailed pairwise t-test, $t(127)=1.385$,

p=0.168). These results provide further evidence that the agency manipulation in Study 1 did shift agency in the predicted manner.

In addition, we used Study 1b to test whether the agency manipulation had an effect on negative affect or trust in the experimenter. After we elicited participants' bids, we measured their negative affect using three items from the Positive and Negative Affect Schedule Expanded Form (PANAS-X)[9]. Participants were asked to indicate the extent to which they felt angry, sad, and upset (in randomized order on a scale from 1="very slightly or not at all" to 5="extremely"). Finally, participants indicated their agreement with the statement, "During this experiment, I trusted that the experimenter would do what the instructions said they would do" (on a scale from 1="do not at all agree" to 5="agree a great deal").

Our analyses revealed there were no significant differences between any of the conditions on any of the measures (two-tailed pairwise t-tests, all ps≥0.126). Additionally, the effect of No Agency on demand for subsequent agency cannot be explained by any of these measures: the coefficient on condition dummy remains significant when they are included in the regression (see Supplementary Table 6).

Supplementary Note 4 – Study 2 Manipulation Check Study (Study 2b)

Similar to Study 1b, the primary goal of Study 2b was to test whether our agency manipulation successfully influences participants' perceived level of agency. The experiment was approved by the UCSD IRB. It complied with all relevant ethical regulations and involved informed consent. The data and analysis code for the study are included in the Supplementary Information files.

UCSD undergraduates (*N*=92) were recruited for the study from a university-wide subject pool. The first part of the study was similar to the first part of Study 2: participants were asked to complete as many anagrams as they could while being exposed to the aversive noise through headphones. Those in the No Agency condition (*N*=46) were told they had to keep their headphones on for the duration of the experiment or be disqualified and forfeit all potential earnings. Participants in the Agency condition (*N*=46) were allowed to remove their headphones at a cost of 50% of their payment.

After completing the anagram task, we measured participants' demand for control using a paradigm similar to the one used in Study 1b. Participants were told they were paired up with another participant in the study. Each submitted a bid (up to 50% of potential bonus earnings) for the ability to choose the winning outcome of a computer-generated coin toss; the person with the lower bid would be assigned the other side of the coin. If the participant won the ability to pick a side and the outcome of the coin toss matched that side, she would receive an additional $10 from which the bid amount would be deducted. If the participant lost the coin toss, no money would be deducted from her earnings. Nothing would be deducted from the earnings of participants with bids lower than their partner's, regardless of the coin flip outcome. Similar to Study 1b, we used bid magnitudes as our measure of demand for agency. We again predicted that participants in the No Agency condition would have greater demand for agency than those in the Agency condition and thus submit higher bids.

All participants kept their headphones on in both conditions. As predicted, bids in the No Agency condition (*M*=30.80) were significantly higher than bids in the Agency condition (*M*=17.46, OLS regression with robust SE, p=0.001). These results indicate that our agency manipulation successfully shifted participants' perceived sense of agency.

The results of our studies offer converging evidence that a lack of agency over an adverse state leads to greater impatience compared to those who have a greater sense of agency over the same state.

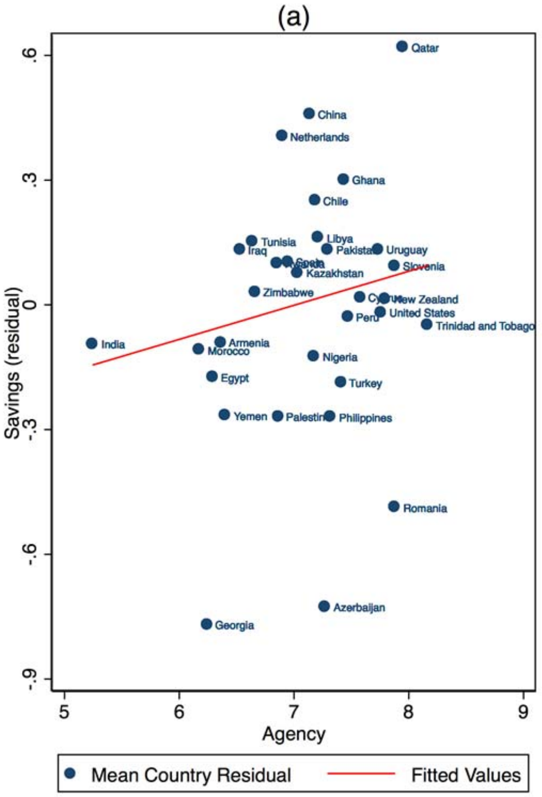

Supplementary Figure 1. World Values Survey results. *N*=24,683 individuals. Panel (a) shows the results of the residual analysis when dividing by country, while Panel (b) shows the results when collapsing across countries.

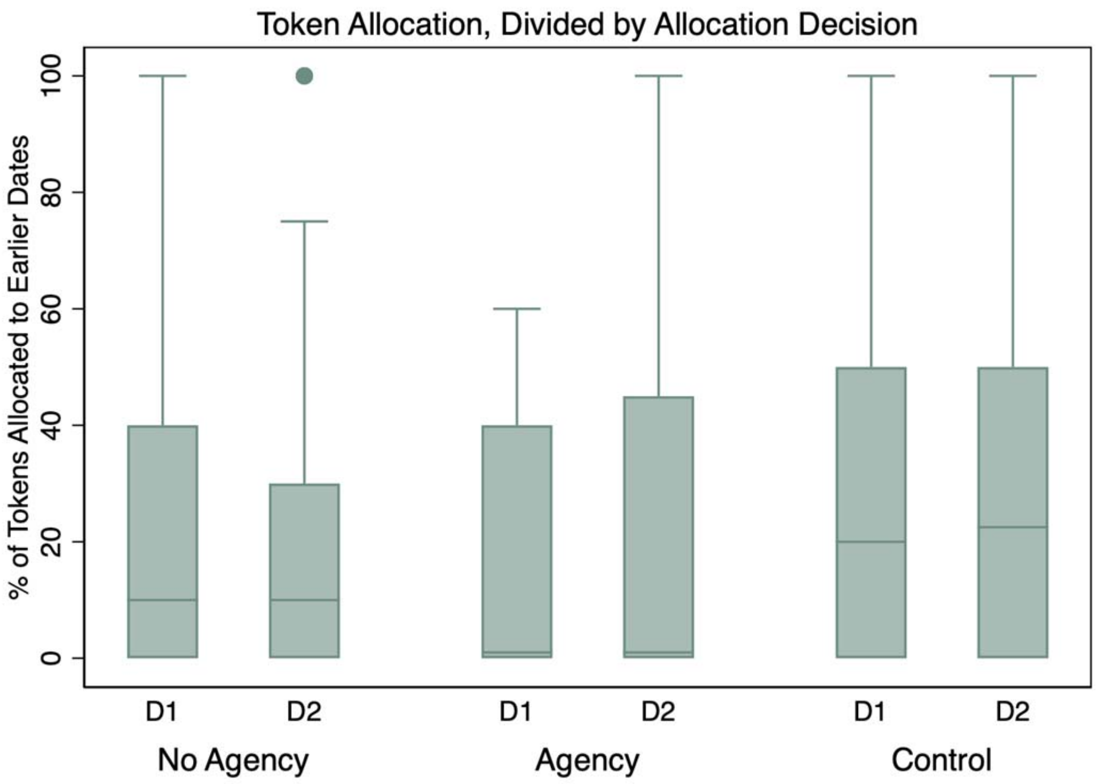

Supplementary Figure 2. Study 1 results: percent of tokens allocated to earlier dates, divided by allocation decision. *N*=211 participants. D1 denotes the first decision participants made ("today" vs. "one week from today"), while D2 denotes the second decision participants made ("one week from today" vs. "two weeks from today"). The box represents the interquartile range (top line: 75th percentile; middle line: median; bottom line: 25th percentile). The upper adjacent value (the top of the whiskers) is the largest observation that is less than or equal to the 75th percentile plus 1.5 times the interquartile range. Dots beyond the whiskers denote observations that extend beyond that point.

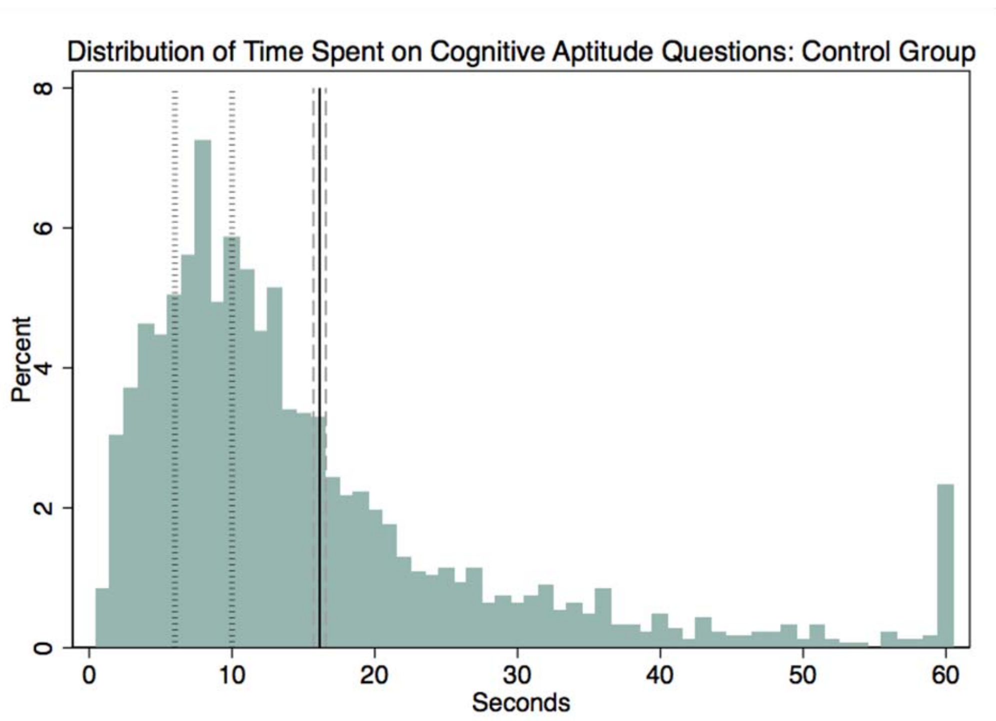

Supplementary Figure 3. Study 1b descriptive statistics: distribution of amount of time Control (No Scarcity) group spent on each cognitive aptitude question. *N*=65 participants. Each Control participant appears in the graph 30 times, once for each cognitive aptitude question. For clarity, the amount of time spent is censored at 60 seconds in this graph. The black solid line denotes mean; the grey dashed lines denote the mean value ±1 SE. The mean and SE are calculated using data prior to censoring. The dotted lines at 10 and 6 seconds denote the amount of time the scarcity group participants had to answer each question in the first and second sets of cognitive aptitude questions, respectively. Compared to participants in the scarcity groups, Control participants spent, on average, 43% more time on the first set of cognitive aptitude questions (Ordinary Least Squares regression with robust SE clustered at the participant ID level, B=7.42 seconds, SE=1.01, p<0.0005) and 59% more time on the second set of cognitive aptitude questions (Ordinary Least Squares regression with robust SE clustered at the participant ID level, B=8.80, SE=1.00, p<0.0005). Treating each participant-question as a separate observation (such that each participant appears in our data 30 times, once for each cognitive aptitude question they answer), we find that 10 seconds corresponds to the 43rd percentile of unconstrained time, while 6 seconds corresponds to the 19th percentile of unconstrained time.

Supplementary Table 1. World Values Survey results.

	OLS with SEs Clustered at Country Level	OLS with Country Fixed Effects	Logit with SEs Clustered at Country Level	Ordered Logit with SEs Clustered at Country Level
Agency	0.014***	0.014***	0.038**	0.027**
	(0.004)	(0.004)	(0.017)	(0.011)
Employed	0.169***	0.169***	0.587***	0.380***
	(0.028)	(0.028)	(0.085)	(0.059)
OECD	0.847***		0.894***	0.317
	(0.075)		(0.247)	(0.194)
OECD*Agency	0.006	0.006	0.027	0.023
	(0.008)	(0.008)	(0.032)	(0.023)
Constant	1.472***	2.356***	-3.642***	
	(0.111)	(0.091)	(0.351)	
N	24381	24381	24381	24381
(Pseudo) R^2	0.20	0.08	0.19	0.09

Outcome variable for columns (1), (2), (4): savings on scale from 1 to 4, with higher numbers indicating more savings. Outcome variable for column (3): binary indicator for whether saved. All standard errors (SEs) are robust and displayed in parentheses. *Employed* is a binary indicator for employment, equaling 1 if the respondent stated being a full time employee, part time employee, or self-employed, and 0 if s/he stated being unemployed. All other responses were coded as missing. *OECD* is a binary indicator for whether the respondent's country is an Organisation for Economic Cooperation and Development (OECD) country. *OECD*Agency* is an interaction variable with *Agency* being treated as continuous. Column (1) Agency: p=0.003. Column (2) Agency: p=0.003. Column (3) Agency: p=0.026. Column (4) Agency: p=0.015. The following control variables were omitted from the table for clarity: income decile indicators, country/year of survey interaction indicators, season of survey indicators, gender indicators, age, number of children, marital status indicators, and education indicators.
* p<0.10, ** p<0.05, *** p<0.01

Supplementary Table 2. Study 1 descriptive statistics.

	No Agency	Agency	Control
Score for Set 1	8.63	8.37	11.32
	(0.24)	(0.22)	(0.27)
Score for Set 2	8.05	8.31	12.28
	(0.25)	(0.27)	(0.33)
Perceived time scarcity (reverse scale)	2.26	2.51	4.59
	(0.12)	(0.15)	(0.14)
N	76	65	72[a]

Means and standard errors of the means (in parentheses). "Set 1" refers to the first set of cognitive aptitude questions, while "Set 2" refers to the second set. Cognitive aptitude question scores are from 0 to 15. Perceived time scarcity is measured on a 7-point scale, with lower numbers indicating greater perceived scarcity.

[a]: *N*=70 participants for the perceived time scarcity variable

Supplementary Table 3. Pooled Study 1 demographics (see Supplementary Notes).

		Scarcity No Agency	Scarcity Agency	Less Scarcity No Agency	Less Scarcity Agency	No Scarcity (Control)	Test statistic (5 groups)	p-value	Test statistic (3 groups)	p-value
Gender	Male	43%	50%	52%	59%	46%	1.48	0.208	0.86	0.423
	Female	57%	50%	48%	41%	54%				
Race	White	80%	80%	82%	83%	81%	0.05	0.995	0.07	0.930
	Black or Af.-Am.	8%	10%	9%	5%	9%				
	Hisp. (non-White)	4%	4%	2%	4%	1%				
	Asian	6%	4%	4%	7%	6%				
	Other/mix	2%	2%	3%	1%	1%				
	No Answer	1%	0%	0%	0%	1%				
Income ($000's)	0-10	14%	16%	17%	16%	7%	4.77	0.310	4.00	0.135
	10-20	16%	20%	29%	15%	12%				
	20-40	32%	21%	24%	28%	32%				
	40-60	19%	23%	22%	25%	28%				
	60-80	9%	10%	8%	7%	4%				
	80-100	6%	3%	6%	4%	10%				
	100-150	2%	3%	4%	5%	7%				
	over 150	2%	2%	0%	0%	0%				
Student	No	89%	83%	83%	85%	85%	0.57	0.685	0.88	0.416
	Yes	11%	17%	17%	15%	15%				
Fluency	No	2%	2%	3%	3%	6%	0.77	0.544	1.56	0.212
	Yes	98%	98%	97%	97%	94%				
Study number(s)		1, 2, 3	1, 2, 3	1, 2	1, 2	3				
N		161	145	93	75	72				

For *Gender, Race, Student,* and *Fluency*, the test statistic and the corresponding *p*-value reflect a one-way ANOVA. For *Income*, which is an ordered variable, the test statistic and the corresponding *p*-value reflect a Kruskal-Wallis test with ties. “Test statistic (5 groups)” tests for differences across all five treatment groups. “Test statistic (3 groups)” tests for differences across the three groups evaluated in the main body of the paper: Scarcity-No Agency, Scarcity-Agency, and Control. *Income* is annual household income, after taxes and deductions. *Fluency* refers to whether the participant reported being fluent in English. See Supplementary Notes for a description of *Study Numbers*. N's in the Agency groups do not include participants who chose to get more time. Percentages may not add up to 100% due to rounding.

Supplementary Table 4. Pooled Study 1 results (see Supplementary Notes).

	(1)	(2)
Scarcity - No Agency	5.6**	5.0*
	(2.5)	(3.0)
Study #2	-4.0	-10.7***
	(2.9)	(4.1)
Study #3	-10.0***	-12.8***
	(2.7)	(3.8)
Second token decision	2.6***	1.8
	(1.0)	(1.2)
Constant	29.5***	33.6***
	(2.2)	(3.3)
N	1087	612
R^2	0.028	0.041
p-value	0.000	0.003

Outcome variable: number of tokens allocated to earlier dates. Ordinary Least Squares regressions with robust standard errors clustered at the participant level. Each participant appears in the regression twice: once for each of the two convex budget token allocations. In (1), all groups other than Scarcity-No Agency are the omitted treatment groups, and Study #1 is the omitted study. In (2), Scarcity-Agency is the omitted treatment group and Study #1 is the omitted study. Studies #1 and #2 are those described in the Supplementary Notes above, and Study #3 is Study 1 of the paper. Column (1) Scarcity - No Agency: p=0.027. Column (2) Scarcity – No Agency: p=0.095. Standard errors in parentheses.

* p<0.10, ** p<0.05, *** p<0.01

Supplementary Table 5. Study 1b descriptive statistics.

	No Agency	Agency (did not exercise agency)	Agency (exercised agency)	Control
Average time per question (Set 1)	10.00	10.00	10.00	17.42
	--	--	--	(0.63)
No. of questions unanswered (Set 1)	1.41	1.75	2.54	0.08
	(0.15)	(0.21)	(0.40)	(0.03)
Average time per question (Set 2)	6.00	6.00	6.67	14.85
	--	--	(0.52)	(0.60)
No. of questions unanswered (Set 2)	1.59	1.73	1.54	0.02
	(0.23)	(0.26)	(0.43)	(0.02)
Sad	1.43	1.42	--	1.40
	(0.09)	(0.11)		(0.11)
Angry	1.30	1.39	--	1.34
	(0.07)	(0.10)		(0.11)
Upset	1.44	1.45	--	1.45
	(0.10)	(0.11)		(0.12)
Trust in experimenter	4.03	4.02	4.54	4.28
	(0.13)	(0.14)	(0.18)	(0.10)
N	79	64	13	65

Means and standard errors of the mean (in parentheses). *Set 1* refers to the first set of cognitive aptitude questions, while *Set 2* refers to the second set. *No. of questions unanswered* refers to the number of questions within the set that participants left blank (out of 15), either because the participant allowed the screen to advance automatically without responding (in the case of the scarcity groups), or because the participant proceeded to the next page without responding (in the case of the Control group).

Supplementary Table 6. Study 1b results.

	(1)	(2)	(3)
No Agency	0.06**	0.06**	0.06**
	(0.03)	(0.03)	(0.03)
Control	0.04	0.04	0.04
	(0.03)	(0.03)	(0.03)
Negative Affect		0.02	
		(0.01)	
Sad			0.01
			(0.03)
Angry			0.00
			(0.03)
Upset			0.01
			(0.03)
Trust In Experimenter		0.00	0.00
		(0.01)	(0.01)
Constant	0.11***	0.10*	0.10*
	(0.02)	(0.06)	(0.06)
N	208	208	208
R^2	0.022	0.031	0.032
p-value	0.077	0.147	0.326

Outcome variable: bid amount ($) to choose the winning side of the coin. Ordinary Least Squares regression with robust SE in parentheses. Agency is the omitted category. Column (1) No Agency: p=0.027. Column (2) No Agency: p=0.026. Column (3) No Agency: p=0.029.
* p<0.10, ** p<0.05, *** p<0.01

Supplementary References

1. Lee, D. S. Training, wages, and sample selection: Estimating sharp bounds on treatment effects. *REStud*, **76**(3), 1071-1102 (2009).
2. Schwarz, N. Self-reports: how the questions shape the answers. *Am Psych*, **54**(2), 93 (1999).
3. Langer, E. J. The illusion of control. *J Personality Social Psych* **32**(2), 311-328 (1975).
4. Brehm, J. W. A theory of psychological reactance. Oxford, England: Academic Press. (1966).
5. Carter, I. The independent value of freedom. *Ethics* **105**(4), 819-845 (1995).
6. Landau, M. J., Kay, A. C., Whitson, J. A. Compensatory control and the appeal of a structured world. *Psych Bull* **141**(3), 694 (2015).
7. Langer, E. J. *The psychology of control*. Beverly Hills: Sage Publications. (1983).
8. Falk, A., Kosfeld, M. The hidden costs of control. *Am Econ Rev* **96**(5), 1611-1630 (2006).
9. Watson, D., Clark, L. A. The PANAS-X: Manual for the positive and negative affect schedule-expanded form. (1999).
