# Supplementary material for: The impact of agency on time and risk preferences
Source: Nat Commun. 2020 May 29;11:2665. doi: 10.1038/s41467-020-16440-0 (PMC7260199; doi:10.1038/s41467-020-16440-0)
Supplement: Supplementary file 2 — Reporting Summary [file 41467_2020_16440_MOESM2_ESM.pdf]

nature research

Corresponding author(s): Ayelet Gneezy

Last updated by author(s): Apr 4, 2020

Reporting Summary

Nature Research wishes to improve the reproducibility of the work that we publish. This form provides structure for consistency and transparency in reporting. For further information on Nature Research policies, see Authors & Referees and the Editorial Policy Checklist .

Statistics

For all statistical analyses, confirm that the following items are present in the figure legend, table legend, main text, or Methods section.

n/a	Confirmed	
☐	☒	The exact sample size (*n*) for each experimental group/condition, given as a discrete number and unit of measurement
☐	☒	A statement on whether measurements were taken from distinct samples or whether the same sample was measured repeatedly
☐	☒	The statistical test(s) used AND whether they are one- or two-sided<br>*Only common tests should be described solely by name; describe more complex techniques in the Methods section.*
☐	☒	A description of all covariates tested
☐	☒	A description of any assumptions or corrections, such as tests of normality and adjustment for multiple comparisons
☐	☒	A full description of the statistical parameters including central tendency (e.g. means) or other basic estimates (e.g. regression coefficient) AND variation (e.g. standard deviation) or associated estimates of uncertainty (e.g. confidence intervals)
☐	☒	For null hypothesis testing, the test statistic (e.g. *F*, *t*, *r*) with confidence intervals, effect sizes, degrees of freedom and *P* value noted<br>*Give P values as exact values whenever suitable.*
☒	☐	For Bayesian analysis, information on the choice of priors and Markov chain Monte Carlo settings
☒	☐	For hierarchical and complex designs, identification of the appropriate level for tests and full reporting of outcomes
☐	☒	Estimates of effect sizes (e.g. Cohen's *d*, Pearson's *r*), indicating how they were calculated

Our web collection on statistics for biologists contains articles on many of the points above.

Software and code

Policy information about availability of computer code

Data collection	Qualtrics XM
Data analysis	Stata 16

For manuscripts utilizing custom algorithms or software that are central to the research but not yet described in published literature, software must be made available to editors/reviewers. We strongly encourage code deposition in a community repository (e.g. GitHub). See the Nature Research guidelines for submitting code & software for further information.

Data

Policy information about availability of data

All manuscripts must include a data availability statement. This statement should provide the following information, where applicable:

- Accession codes, unique identifiers, or web links for publicly available datasets
- A list of figures that have associated raw data
- A description of any restrictions on data availability

All data produced by the authors are available in the supplementary information files and on the Open Science Framework at osf.io/hfz98/ . The data for the World Values Survey is available at http://www.worldvaluessurvey.org/WVSDocumentationWV6.jsp .

Field-specific reporting

Please select the one below that is the best fit for your research. If you are not sure, read the appropriate sections before making your selection.

☐ Life sciences ☒ Behavioural & social sciences ☐ Ecological, evolutionary & environmental sciences

Behavioural & social sciences study design

All studies must disclose on these points even when the disclosure is negative.

Study description	Both studies were quantitative experimental
Research sample	World Values Survey (WVS): These data were collected by social scientists interested in individuals' beliefs and values and the relationship between those values and their social and political characteristics. The Wave 6 (2010-2014) data span 61 countries across the world and contain 86,272 observations. The data were gathered through in-person interviews in the native language of the respondents. The WVS uses either full probability or quota sampling and takes care to minimize non-response. The sample is representative of the inhabitants of each country sampled. The data include several hundred variables capturing basic respondent demographics (age, gender, health, employment status, income, savings, etc.), sociological / cultural beliefs (religious beliefs, political views, etc.), and psychological measures (feelings of loneliness, happiness, agency, etc.).<br><br>Study 1: The goal of the study was to identify the causal relationship between scarcity and agency on time and risk preferences. Participants were recruited through Amazon's Mechanical Turk. The participant group was approximately 51% female, 82% white, 96% fluent in English, 85% non-students, and with an average income of approximately $50,000/year. The sample is roughly representative of the population on Mechanical Turk, though Mechanical Turk participants are in general more likely to be White, highly educated, and liberal than the average American. We used Mechanical Turk participants to maximize our sample size, given our time and financial constraints.<br><br>Study 2: The goal of the study was to identify the causal relationship of an environmental stressor on participants' time preferences. Participants were undergraduates at the University of California, San Diego (UCSD). Demographic information was not collected. The sample is more likely to be familiar with psychological / behavioral economics studies and concepts than the average UCSD student, given the distribution of majors in the participant pool. Because it was important to be able to monitor whether participants actually took off their headphones, the study could not be completed remotely. Thus, we completed it using an in-person lab.
Sampling strategy	World Values Survey: We used all observations in the dataset that were not missing information for the variables of interest.<br><br>Study 1: Any Mechanical Turk worker who signed up for the experiment, was in the United States, had a 90% or higher approval rating, and agreed to the consent form was allowed to participate. Sample size was determined based on pilots and a power calculation (Lenth, R. V. (2006-9). Java Applets for Power and Sample Size [Computer software]. Retrieved from http://www.stat.uiowa.edu/~rlenth/Power), balanced with the financial cost of running studies. These power calculations revealed that for the effect sizes and standard deviations of the pilots, we could achieve 80% power with approximately 75 participants per cell and repeated measures.<br><br>Study 2: A target sample size was determined prior to running the study. The paper on which we based our manipulation (Alloy & Abramson, 1982) had used 120 participants, while the paper on which we model our dependent variable (Kirby, Petry, & Bickel, 1999) had used 116 participants. The experimenters estimated that a comparable sample size (in our case, a recruited sample of 115) would provide sufficiently high power while keeping experimenter time and effort costs relatively low.
Data collection	World Values Survey: In-person interviews were used.<br><br>Study 1: Data were collected remotely online. The study was run through the survey platform Qualtrics. The researchers were not present during data collection and had no way of interacting with the participants, other than to provide bonuses at the end of the study.<br><br>Study 2: Data were collected through the survey platform Qualtrics. Participants completed the survey on computers in the lab. Each session contained 1 to 8 participants. The same experimenter (the second author, AI) was always present for all the sessions. He was not blind to the study hypothesis but was blind to the experimental condition of the participants.
Timing	World Values Survey: 2010 - 2014<br><br>Study 1: 10/1/15-10/11/15<br><br>Study 2: 5/14/12-5/15/12
Data exclusions	World Values Survey: No observations were excluded, other than those that were missing data for the variables of interest.<br><br>Study 1: Four participants were excluded for exercising their increased agency. We did not collect dependent variable measures for these participants.<br><br>Study 2: Two participants were excluded for exercising their agency. These participants still completed the dependent variable measures. Including them in the analysis does not substantively change any conclusions. For the parametric analysis, four participants were excluded for always choosing either the first (smaller-sooner) or second (larger-later) reward for each of the 27 time preferences questions, as such responses yield non-rationalizable time preferences. These four participants are still included in the non-parametric analysis.<br><br>Exclusion criteria for both studies was pre-established.

Non-participation	Study 1: Three participants began but failed to complete the study. It is not clear why they abandoned the study.<br><br>Study 2: No participants failed to complete the study once beginning it.
Randomization	Both studies: Participants were randomly assigned to treatment groups using the Qualtrics randomization feature.

Reporting for specific materials, systems and methods

We require information from authors about some types of materials, experimental systems and methods used in many studies. Here, indicate whether each material, system or method listed is relevant to your study. If you are not sure if a list item applies to your research, read the appropriate section before selecting a response.

Materials & experimental systems

n/a	Involved in the study
☒	☐ Antibodies
☒	☐ Eukaryotic cell lines
☒	☐ Palaeontology
☒	☐ Animals and other organisms
☐	☒ Human research participants
☒	☐ Clinical data

Methods

n/a	Involved in the study
☒	☐ ChIP-seq
☒	☐ Flow cytometry
☒	☐ MRI-based neuroimaging

Human research participants

Policy information about studies involving human research participants

Population characteristics	See above
Recruitment	Study 1: Participants were recruited from Amazon Mechanical Turk. The task was advertised as a study on decision-making with keywords "survey" and "demographics." Participants completed the study for a base payment and the possibility of an additional bonus payment. Participants had to be in the United States and had have at least a 90% approval rating on Mechanical Turk. Given the reason that most Mechanical Turk workers work (earning income), participants were likely lower income and/or more liquidity constrained than the median American, which may have increased the strength of the manipulation. Thus, it is possible that less financially constrained participants would react differently to our manipulation. In addition, participants were likely more familiar with psychological and behavioral economics survey measures than the average person, which may have made it easier for them to understand the time preferences elicitation. Less experienced or less educated individuals may have greater difficulty understanding the measure.<br><br>Study 2: Participants were recruited from the University of California, San Diego research participant pool. Undergraduates came to the lab in exchange for class credit. Participants were more likely to be younger, highly educated, and familiar with psychological or behavioral science experiments than the average person. We do not believe that the self-selection here poses any major concern for the interpretation of our results.
Ethics oversight	Study 1: The study was approved by the Carnegie Mellon University Institutional Review Board<br><br>Study 2: The study was approved by the University of California San Diego Institutional Review Board

Note that full information on the approval of the study protocol must also be provided in the manuscript.
