# Supplementary material for: The impact of agency on time and risk preferences
Source: Nat Commun. 2020 May 29;11:2665. doi: 10.1038/s41467-020-16440-0 (PMC7260199; doi:10.1038/s41467-020-16440-0)
Supplement: Supplementary file 3 — Description of Additional Supplementary Files [file 41467_2020_16440_MOESM3_ESM.pdf]

Description of Additional Supplementary Files

File Name: Supplementary Data 1
Description: Data for Study 1

File Name: Supplementary Data 2
Description: Data for Study 1b

File Name: Supplementary Data 3
Description: Main data for Study 2

File Name: Supplementary Data 4
Description: Discount rates data for Study 2

File Name: Supplementary Data 5
Description: Data for Study 2b

File Name: Supplementary Software 1
Description: Analysis code for World Values Survey analysis

File Name: Supplementary Software 2
Description: Analysis code for Study 1 analysis

File Name: Supplementary Software 3
Description: Analysis code for Study 1b analysis

File Name: Supplementary Software 4
Description: Analysis code for Study 2 analysis

File Name: Supplementary Software 5
Description: Analysis code for Study 2b analysis
